# Albumin-Enriched Fibrin Hydrogel Embedded in Active Ferromagnetic Networks Improves Osteoblast Differentiation and Vascular Self-Organisation

**DOI:** 10.3390/polym11111743

**Published:** 2019-10-24

**Authors:** Galit Katarivas Levy, John Ong, Mark A. Birch, Alexander W. Justin, Athina E. Markaki

**Affiliations:** 1 Department of Engineering, University of Cambridge, Trumpington Street, Cambridge CB2 1PZ, UK; jo401@cam.ac.uk (J.O.); awj27@cam.ac.uk (A.W.J.); 2 Division of Trauma and Orthopaedic Surgery, Addenbrooke’s Hospital, Hills Road, Cambridge CB2 2QQ, UK; mab218@cam.ac.uk

**Keywords:** ferromagnetic fibre network, human albumin, fibrin hydrogel, human foetal osteoblasts, human endothelial cells

## Abstract

Porous coatings on prosthetic implants encourage implant fixation. Enhanced fixation may be achieved using a magneto-active porous coating that can deform elastically in vivo on the application of an external magnetic field, straining in-growing bone. Such a coating, made of 444 ferritic stainless steel fibres, was previously characterised in terms of its mechanical and cellular responses. In this work, co-cultures of human osteoblasts and endothelial cells were seeded into a novel fibrin-based hydrogel embedded in a 444 ferritic stainless steel fibre network. Albumin was successfully incorporated into fibrin hydrogels improving the specific permeability and the diffusion of fluorescently tagged dextrans without affecting their Young’s modulus. The beneficial effect of albumin was demonstrated by the upregulation of osteogenic and angiogenic gene expression. Furthermore, mineralisation, extracellular matrix production, and formation of vessel-like structures were enhanced in albumin-enriched fibrin hydrogels compared to fibrin hydrogels. Collectively, the results indicate that the albumin-enriched fibrin hydrogel is a promising bio-matrix for bone tissue engineering and orthopaedic applications.

## 1. Introduction

Total hip replacement (THR) is one of the most common surgeries performed in the world [1]. During the procedure, the degenerating femoral head, often with the acetabulum, is replaced with metal or ceramic prosthetic implants to reduce joint pain, enhance joint function and improve quality of life for patients [2]. While THR is likely to be a ‘life-long’ implant for patients aged 65 or older, for younger patients (50–54 years), there is a 35% probability of undergoing a revision within their lifetime [3]. By 2030, 52% of primary THRs are projected to be implanted in patients younger than 65 years, with the highest increase in patients aged 45–55 years [3]. As a consequence, the number of revisions is expected to increase significantly, and this, in turn, would increase the socio-economic burden on already stretched healthcare systems globally [4]. The most common indication for a THR revision is aseptic loosening and seldom due to mechanical failure of the prosthesis itself [5,6]. Aseptic loosening is in part due to the poor fixation and integration of the bone with a prosthesis. Currently, there is an increasing trend toward cementless THRs which contain permeable porous coatings, especially in younger and more active patients placing additional demands on the device performance [1,7]. Highly porous coatings improve permeability along the bone-prosthesis interface and facilitate the transport of oxygen and nutrients which is dependent on diffusion gradients in blood [8]. It then allows the in-growth of bone into the edges of prostheses to improve fixation.

**Figure 1:**
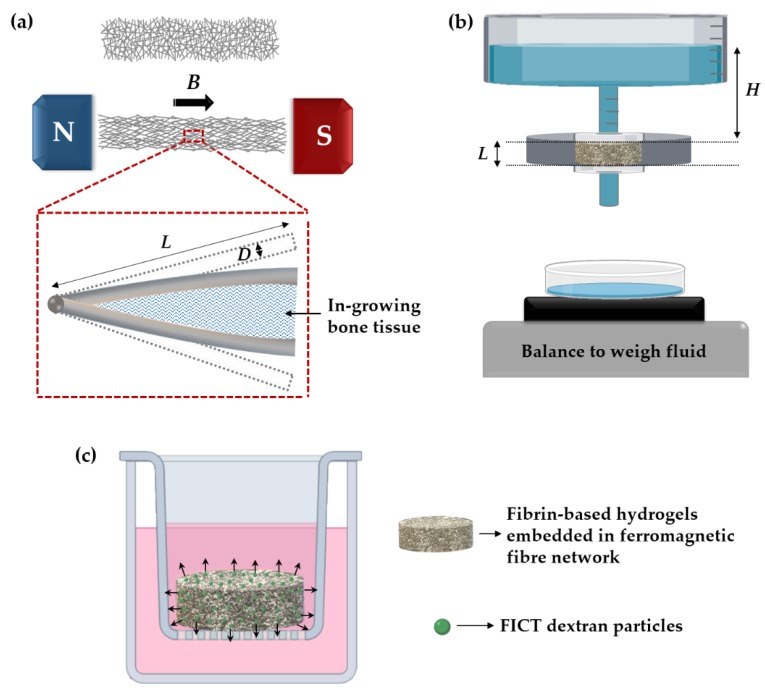
(**a**) Schematic representations of the elastic deformation of a fibre network under a magnetic field *B*. Also shown is the deflection of a bonded pair of fibres deforming in-growing bone tissue; Schematic of the set-up employed for measuring: (**b**) specific permeability; (**c**) FICT-dextran diffusion of the scaffolds.

A porous magneto-active layer, made of slender ferromagnetic fibres bonded together at cross-over points, has been proposed for use as a THR implant coating [9,10]. The purpose of this layer is to grow healthy periprosthetic bone through the application of an external magnetic field of clinical magnitude. When a magnetic field is applied, the fibres deflect as they become fully magnetised, imposing strains to any (compliant) matrix material filling the inter fibre spaces (Figure 1a). A strong candidate for the fibre material is 444 ferritic stainless steel due to its biocompatibility [11]. It is a soft magnetic material (i.e., has low coercivity and remanence) and exhibits a relatively high magnetisation. Our recent in vitro work [12] has demonstrated that daily magneto-mechanical actuation (0.3–1.1 Tesla at 0.2 Hz for 5 h) for two weeks could enhance mineralisation, extracellular matrix (ECM) production, and upregulate genes and proteins involved in osteogenesis. However, we recognise that wound healing and bone regeneration in vivo is more complex and extends beyond the response of a single cell type to mechano-transduction.

One of the most important factors that determine the success of a cementless prosthesis is the reconstruction of healthy bone tissue around the prosthesis [13], however for this to be achieved problems with inadequate vascularisation must first be overcome [14]. In bone, microvessels are essential for bone formation, metabolism, healing, and remodelling [15]. Inadequate vascularisation at the site of implantation results in insufficient oxygen and nutrition supply as well as leads to the accumulation of waste products. All these contribute to hypoxic injury and eventually cell death. Therefore, the development of functional blood vessels in bone tissue scaffolds are vital to successful therapeutic outcomes [16]. A strategy for promoting osseointegration and vascularisation in porous implants involves the infusion of the structure with a suitable bio-matrix that will guide and direct proliferation and differentiation of migratory or encapsulated progenitor cells towards the appropriate lineage [14,17]. Natural protein–based polymers derived from blood such as fibrin and albumin offer several benefits over synthetic materials because their properties mimic the ECM of the damaged tissue more closely [18,19]. Therefore, to recapitulate the in vivo environment of wound healing and bone regeneration more closely, we hypothesised that a hydrogel could be created from blood components to accelerate bone and microvasculature growth since blood is present at both the time of bone injury and regeneration.

Albumin is the most abundant human plasma protein, which is involved in a variety of roles related to cell survival and regeneration [19]. It transports many small molecules such as calcium and magnesium and combines with heavy metals to prevent toxicity. It has been shown that osteoblast cells are capable of albumin production and this increases locally after bone fracture [20,21,22]. Several studies have shown that albumin is an efficient coating for bone-related biomaterials associated with increasing seeding efficiency, cell proliferation, and calcium deposition in vitro [23,24,25] and bone remodelling in vivo [24,25,26,27,28]. However, the use of albumin hydrogels in bone tissue engineering remains under-utilised [29]. Fibrin is one of the most promising biopolymers used for bone and cardiovascular tissue engineering applications due to a combination of excellent biocompatibility, biodegradability, intrinsic bioactivity, and many other unique characteristics [30,31]. Fibrin is naturally formed by the enzymatic polymerisation of fibrinogen with thrombin [32]. It is an FDA approved material that can also bind cell-derived growth factors and can be isolated easily from a patient’s blood, enabling the fabrication of an entirely autologous scaffold [30]. Therefore, the literature on the use of fibrin in tissue engineering applications is extensive and continues to evolve [30]. Fibrin hydrogels form a 3D network of branching fibres [33], which can be described by variables such as the thickness of the fibres, number of branch points, porosity, and permeability of the gels [34]. This structure can vary extensively with changes in the polymerisation process such as varying fibrinogen and thrombin concentrations, pH, temperature, and the presence of salt concentration or plasma proteins [30]. These changes can dramatically affect cell behaviour and need to be investigated carefully. While fibrin and fibrin-based biomaterials have been successfully used for tissue engineering [15], no study has evaluated the potential of the combination of albumin and fibrin for bone regeneration.

In this study, novel xeno-free hydrogels were synthesised from two major components of human blood—albumin and fibrin. The hydrogels (fibrin and albumin/fibrin) were then cast into a magneto-active layer aimed for THR applications in order to investigate the effect on osteogenesis and vessel-like formation. An investigation was carried out on the fibrous structure and Young’s modulus of the free-standing hydrogels followed by measurements of the specific permeability and FITC-dextran diffusion of hydrogel-impregnated fibre networks. Cellular responses were assessed in terms of matrix mineralisation, and gene expressions against osteogenic and angiogenic markers. Human endothelial cell self-organisation into vessel-like structures in co-culture with human foetal osteoblasts was characterised.

## 2. Materials and Methods

### 2.1. Ferromagnetic Fibre Networks

Solid-state sintered stainless-steel fibre networks made of 444 ferritic stainless steel (Nikko Techno Ltd., Tokyo, Japan) were used in this study. The fibre networks were produced by shaving 60 µm fibres off a 100 µm thick 444 foil, which led to a rectangular cross-sectional shape. The networks contain ~15 vol% of fibres with a mean fibre inclination angle between the fibre axis and the through-thickness direction of 81.87 ± 0.21°. Details on the manufacturing of this network and information on its fibre architecture have been discussed in previous studies [11,35,36,37]. For all experiments, discs of 10 mm diameter were cut out from a sheet of ~1 mm thick using a punch press. The discs were ultrasonically cleaned for 15 min sequentially in acetone, ethanol and ultrapure water, dried in air at room temperature followed by sterilisation at 126 °C for 20 min using a Prestige Medical™ Classic Autoclave (Prestige Medical, Blackburn, UK).

### 2.2. Cell Co-Culture

Human umbilical vein endothelial cells (HUVECs) labelled with green fluorescent protein (GFP) were obtained from Cellworks, Buckingham, UK (ZHC-2402) and were cultivated in EGM-2 medium, supplemented with a bullet kit containing fetal bovine serum (FBS), hydrocortisone, hFGF-β, VEGF, R3-IGF1, hEGF, GA-1000, and heparin (PromoCell, Heidelberg, Germany, C-22111). Foetal human osteoblasts (fHObs), obtained from the European Collection of Cell Cultures (Public Health England, Porton Down, UK, 406-05f), were cultivated in McCoy’s 5A medium (Gibco™, Thermo Fisher Scientific, London, UK, 16600082), supplemented with 10% FBS (Invitrogen, Thermo Fisher Scientific, London, UK, 10108-157), 1% (*v*/*v*) Penicillin-Streptomycin (Sigma–Aldrich, Haverhill, UK, P4333), and 50 mg·mL^−1^ L-Ascorbic Acid Phosphate Magnesium Salt (FUJIFILM Wako Chemical Corporation, Richmond, VA, USA, 013-19641). HUVECs and fHObs in the fourth passage were used for all experiments. In order to generate vascular-like networks, co-cultures of HUVECs and fHObs were used at a ratio of 4:1 (320,000 and 80,000 cells per scaffold, respectively) in a co-culture medium (4:1 mixture of the two respective cell lines).

### 2.3. Fabrication of Hydrogels and Hydrogel-Impregnated Fibre Networks

Fibrin (F) and albumin-enriched fibrin (AF) hydrogels were produced by combining human fibrinogen (Merck Chemical Ltd., Nottingham, UK, 341576), human thrombin (Sigma–Aldrich, Haverhill, UK, T6884-100UN), human albumin (Sigma–Aldrich, Haverhill, UK, A1653), and co-culture medium. Albumin powder was dissolved in co-culture medium and the solution was filtered using a 0.45 μm syringe microporous filter. The final fibrinogen and albumin concentration in the gels was 10 mg·mL^−1^ (5 U·mL^−1^ thrombin) and 10 mg·mL^−1^ respectively. We chose to add 10 mg·mL^−1^ of albumin to the fibrin hydrogel since it was reported [38] that low concentrations of albumin have a minor effect on the fibre assembly in the fibrin polymerisation process. 444 fibre networks were impregnated with F and AF hydrogels, designated as 444_F and 444_AF respectively. Sterile 444 fibre network samples were placed onto sterile hydrophobic PTFE (polytetrafluoroethylene) membranes (5 μm pore size, Fisher Scientific, 10676741). Then, 30 µL of albumin was added to 32 µL of co-culture medium with or without cells (for fibrin hydrogels 30 µL of co-culture medium was added). The mix was combined with 7.5 µL human thrombin and seeded onto the fibre networks. Then, 12 µL of human fibrinogen was added to each scaffold, the mixture was gently pipetted, and the scaffolds were incubated for 2 h to polymerise. They were then each transferred to a well of a 24-well plate, covered with 1.5 mL of co-culture medium. To induce differentiation, 10 nM dexamethasone (Sigma–Aldrich, Haverhill, UK, D2915) and 10 mM β-glycerophosphate (Thermo Fisher Scientific, London, UK, 10424701) were added to the culture medium after two days in culture in accordance with previous work [39]. The medium was replenished every other day.

### 2.4. Scanning Electron Microscopy and Morphometric Analysis of Hydrogel-Impregnated Network Structure

The networks were fixed in 10% formalin (VWR International, Lutterworth, UK, 11699455) under a fume hood overnight. They were then washed thrice with PBS and dehydrated in increasing concentrations of ethanol: deionized water mixtures; 30%, 50%, 70%, 80%, 90%, 95%, and 100% ethanol. The last step involving 100% ethanol was repeated twice. Networks were submerged in each ethanol: deionized water mixture for 30 min. For drying, dehydrated networks were submerged in increasing concentrations of hexamethyldisilazane (HMDS): ethanol mixtures under a fume hood at 33.3%, 66.6%, and 100% HMDS, respectively, for 40 min each. The last step (100% HMDS) was repeated twice. Thereafter, networks were left submerged in 100% HMDS overnight in a fume hood until completely dry the following day. After drying, they were coated with a gold–palladium mixture using a Polaron sputter coater. Hydrogel-impregnated networks (*n* = 3 samples from each group) were examined using a scanning electron microscope (Zeiss EVO^®^ *LS 15*). The fibre diameter and pore area of the dried hydrogels were measured using the DiameterJ plugin in ImageJ (an open-source nanofiber diameter measurement [40]) from five randomly selected areas (9.25 × 7 mm^2^), from each sample.

### 2.5. Hydrogel Mechanical Testing

Fibrin and albumin-enriched fibrin hydrogels were tested under compression using a customised ‘see-saw’ set-up as described previously [41]. Briefly, the set-up has a central pivot resting on a frictionless support base that perfectly balances the free extending arms on either side. A flat loading platen was fixed beneath the end of the arm. The load was ramped up at a constant rate (~1.67 × 10^−4^ N·s^−1^) by placing 0.5 gr aluminium discs (~0.005 N) on the end of the arm. The resulting displacement was monitored using a bi-axial laser micrometre (resolution of ±3 µm). The through-thickness Young’s modulus was measured from the tangent slope of the stress-strain curve (up to 5% strain). Five samples were tested for each hydrogel group.

### 2.6. Specific Permeability of Hydrogel-Impregnated Networks

The specific permeability was measured using a constant pressure gradient method, as shown in Figure 1b and as described previously [41]. Briefly, the rig allows small pressure differences to be imposed across the scaffold, defined by the hydrostatic head of water (∆*P* = *ρ*·*H*·*g*) since the bottom of the scaffold is exposed to the atmosphere. The pressure was held constant across the scaffold (with thickness *L*), and the volumetric flow rate (*Q*) of distilled water through the scaffold was measured (from the mass of water passing through the scaffold in a given time). This mass was measured, using an analytical balance with a precision of 1 mg, and converted to volumetric flow using the water density (*ρ* = 0.998 Mg·m^−3^). From *Q*, the sectional area (*A*) and the pressure gradient, ∆*P/L*, the specific permeability, *κ*, was calculated using Darcy’s Law,

(1) $$\kappa \left[m^{2}\right]=\frac{\eta \left[Pa\cdot s\right]\cdot Q\left[m^{3}\cdot s^{-1}\right]\cdot L\left[m\right]}{A\left[m^{2}\right]\cdot \Delta P\left[Pa\right]}$$

in which *η* is the dynamic viscosity of the water (taken as 8.9 × 10^−4^ Pa·s). A total of 5 scaffolds per group (10 mm diameter, 1 mm height) were used. The pressure gradients created in the samples during these experiments were about 80 Pa·mm^−1^.

### 2.7. FITC-Dextran Diffusion

In order to investigate the release kinetics of proteins in the 444_F and 444_AF scaffolds [42], 100 µg·mL^−1^ of FITC-dextran 70 kDa (Sigma–Aldrich, Haverhill, UK, 46945) particles were incorporated into the hydrogels in the preparation stage. After 2 h of incubation, the scaffolds were placed in inserts in 12-well plates (Figure 1c). Each scaffold (10 mm in diameter and 1 mm in height) had an average total surface area of 110 mm^2^. The well plates were covered with aluminium foil and kept in the incubator. After 24 h, the medium from each scaffold was pipetted and 100 µL samples were placed in triplicates to a black 96-well microplate. Fluorescence of FITC-dextran particles (excitation 492 nm, emission 518 nm) was measured on a FLUOstar^®^ Omega plate reader (BMG Labtech, Aylesbury, UK). A total of five scaffolds for each group were tested. The concentration of the dextran particles was determined using a standard curve with a known concentration of the particles.

### 2.8. Vascular Analysis Using AngioTool

Formation of ‘vascular-like structure’ (preliminary endothelial cell structures) was investigated out using the AngioTool [43] on images obtained from a Zeiss Axio-Observer.Z1 fluorescence microscopy after 16 and 21 days of culture. The images were analysed for vessel coverage, total vessel length and total branching points. Five randomly selected areas (2.7 × 2.7 mm^2^), were analysed from each scaffold (*n* = 3 samples from each group).

### 2.9. Cell Mineralisation

Alizarin Red Staining (Sigma–Aldrich, Haverhill, UK, A5533) was used to evaluate mineralisation activity at days 16 and 21 of culture. The scaffolds were washed with phosphate-buffered saline (PBS) and fixed in 4% (*v*/*v*) formaldehyde at room temperature for 30 min. After washing with excess distilled water (dH_2_O), Alizarin Red solution (2% *w*/*v* in dH_2_O adjusted to pH 4.2 using 0.5% ammonium hydroxide) was used to cover the samples for 30 min. After aspiration of the unincorporated dye, the samples were washed thoroughly with dH_2_O. The samples were visualised using a Zeiss Axio-Observer.Z1 fluorescence microscope (fluorescence emission 580 nm). In order to extract the stained minerals from the scaffolds (*n* = 3 samples from each group), 800 μL of 10% acetic acid was added to each sample followed by 30 min incubation at room temperature as described before [44]. The Alizarin Red dye concentration from the samples was measured using the FLUOstar^®^ Omega plate reader (BMG Labtech, Aylesbury, UK) at 405 nm. An Alizarin Red staining standard curve was established with a known concentration of the dye.

### 2.10. Real-Time Polymerase Chain Reaction (RT-PCR)

At day 16 and 21 of culture, the total RNA was extracted from the cell-seeded scaffolds using RNeasy Protect Mini Kit (Qiagen, Manchester, UK, 74124) according to the manufacturer’s instructions. Complementary deoxyribonucleic acid (cDNA) was synthesised by reverse transcriptase–polymerase chain reaction (RT-PCR) by using QuantiTect^®^ Reverse Transcription Kits (Qiagen, Manchester, UK, 205311) according to the manufacturer’s instructions. RT-PCR was conducted using the QuantiFast SYBR Green PCR Kit (Qiagen, Manchester, UK, 204056) with the following primers: Glyceraldehyde-3-phosphate dehydrogenase (GAPDH), human alkaline phosphates (ALP), collagen type 1α1 (COL1A1), osteocalcin (OCN), bone morphogenetic protein 2 (BMP-2, QT00012544), vascular endothelial growth factor (VEGF), von-Willebrand factor (vWF), angiopoietin 1 (Ang-1), and angiopoietin 2 (Ang-2), which amplify transcripts characteristic of endothelial cells and osteoblasts. Primer sequences are listed in Table 1. The cycle conditions were performed with a 5 min activation step at 95 °C followed by 40 cycles with a 10 sec at 95 °C denaturation and 30 min at 60 °C extension step. GAPDH expression served as an internal control. 3 samples from each group for every time point. Relative expression was calculated using the 2^−ΔΔCT^ method according to Livak and Schmittgen [45]. Results were presented as fold change expression normalised to the 444 fibre network impregnated with fibrin hydrogel to determine the effect of albumin on the expression of osteogenic and angiogenic gene markers.

### 2.11. Statistical Analysis

The results are presented as mean  ± standard error. Data were analysed and graphed using GraphPad Prism version 8.2.1 for Windows (GraphPad Software, San Diego, CA, USA). An unpaired *t* test was performed to determine the differences between 444_AF and 444_F scaffolds in permeability, diffusion, mineralisation and RT-PCR analyses. Young’s modulus difference was evaluated by unpaired *t* test between the albumin enriched fibrin and fibrin hydrogels. The threshold for statistical significance was set at a value of *p* < 0.05.

## 3. Results and Discussion

### 3.1. Morphometric Analysis of Hydrogel-Impregnated Network Structure

**Figure 2:**
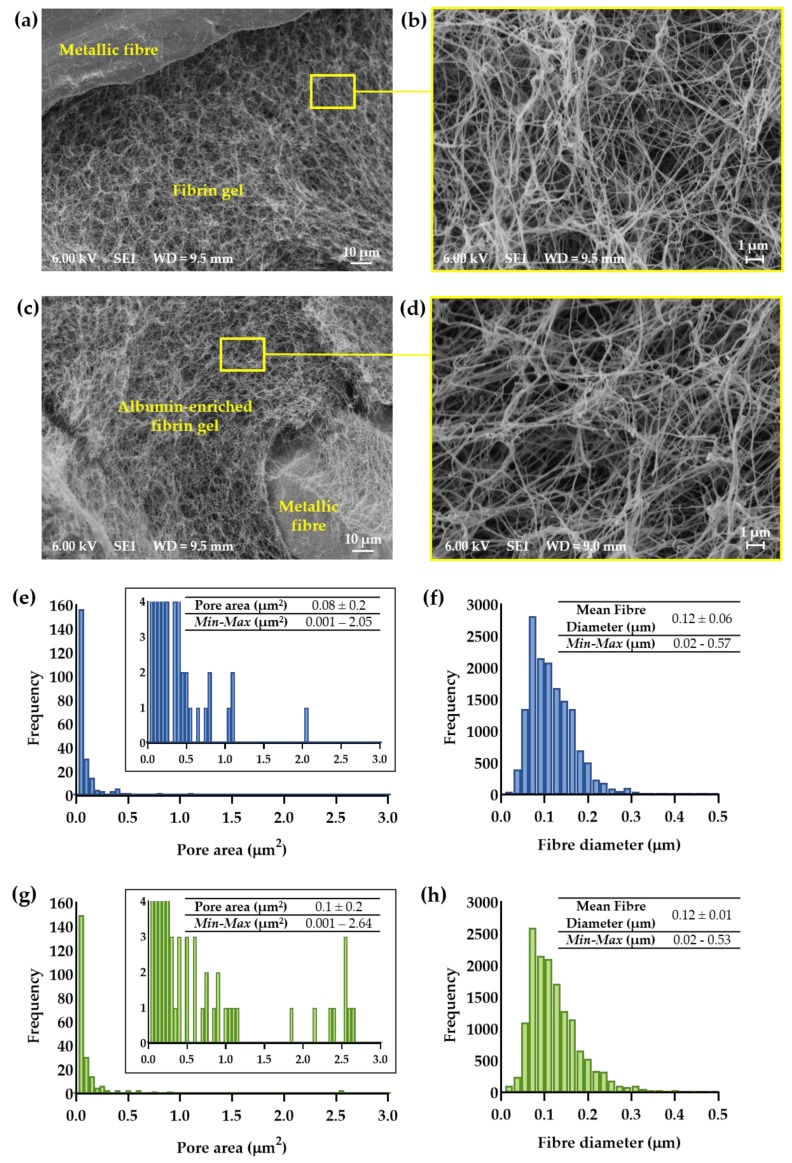
Typical scanning electron micrographs showing top views of 444 networks impregnated with (**a**,**b**) fibrin (444_F) and (**c**,**d**) albumin-rich fibrin (444_AF) hydrogels; Distributions of pore area and fibre diameter for: (**e**,**f**) 444_F and (**g**,**h**) 444_AF as measured by DiameterJ (*n* = 3 samples from each group and five randomly selected areas from each sample).

Scanning electron micrograph of the 444_AF and 444_F ferritic stainless steel fibre networks are shown in Figure 2a–d. It can be seen that the hydrogels have filled the inter-fibre spaces of the networks (Figure 2a,c) and that they consist of a typical 3D matrix composed of elongated branching fibres (Figure 2b,d). Figure 2e–h shows that the mean fibre diameter was very similar for both hydrogels with 95% of the measured values between 0.05 and 0.2 µm. Both hydrogels showed a wide distribution of pore areas as expected for fibrin-based hydrogel. However, AF hydrogels showed a larger proportion of pore areas above 0.1 µm^2^ compared to the F hydrogels (5% vs. 1.7%), suggesting that the albumin-enriched fibrin hydrogels have a higher number of larger pores compared to the fibrin. This might be related to the presence of albumin in the fibrin formation process. It is well known that fibrin structure can vary extensively with changes in the conditions of polymerisation such as fibrinogen and thrombin concentrations, pH, temperature and the presence of salt concentration or plasma proteins [30]. Though albumin does not bind to the fibrinogen and thrombin [46,47], fibrin hydrogels formed with albumin differ structurally from those formed without by increasing the pore size [48]. According to Torbet’s study on fibrin assembly in human plasma [38], the addition of albumin to fibrin increased the fibre thickness and porosity in the fibrin structure. The most significant changes in those parameters were observed in higher albumin concentrations (36–78 mg·mL^−1^) compared to lower concentrations (0–18 mg·mL^−1^). In this study, the differences were observed only in the pore area which can be explained by the higher concentration of fibrinogen (10 mg·mL^−1^ vs. 1 mg·mL^−1^) and thrombin (5 U·mL^−1^ vs. 0.12 U·mL^−1^) compared to Torbet’s study.

### 3.2. Hydrogel Mechanical Testing

**Figure 3:**
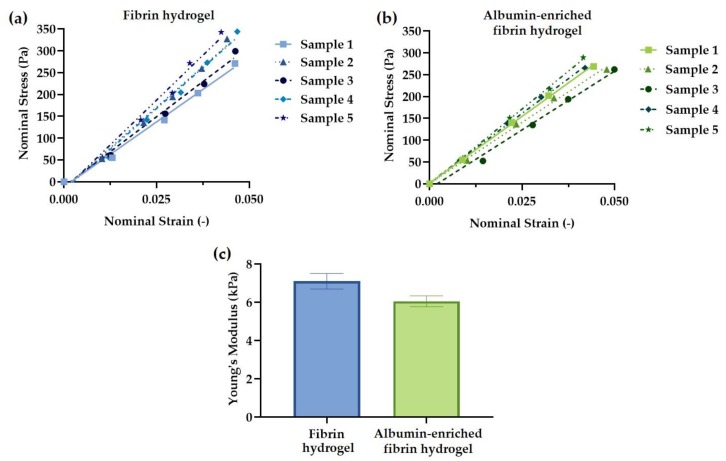
Stress-strain curves for (**a**) fibrin (F) and (**b**) albumin-rich fibrin (AF) hydrogels; (**c**) Young’s modulus was measured from the tangent slope of the stress-strain curves (*n* = 5 samples from each group). No statistically significant differences between the two hydrogels were observed from the unpaired *t* test (*p* = 0.07).

The mechanical microenvironment regulates signal transduction in endothelial cells and thereby controls the vascular morphogenesis [42]. In order to evaluate the addition of albumin to the fibrin hydrogel, the Young’s modulus of free-standing hydrogels was measured using a customised “see-saw” set-up. Figure 3a,b shows the stress-strain response for up to 5% strain. The Young’s modulus was calculated from the tangent slope of the stress-strain curves and the values obtained are shown in Figure 3c. The Young’s modulus for F and AF hydrogels were measured to be 7.1 ± 0.4 kPa and 6.0 ± 0.3 kPa, respectively. No statistically significant differences were observed from the unpaired *t* test (*p* = 0.07). Therefore, the addition of albumin to the fibrin hydrogel did not have a notable effect on the stiffness of the fibrin hydrogel.

### 3.3. Specific Permeability and Diffusion of Hydrogel-Impregnated Networks

**Figure 4:**
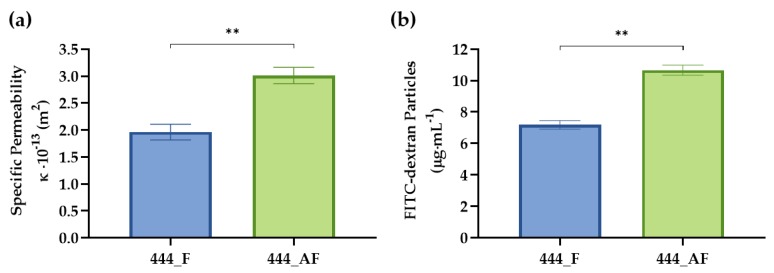
(**a**) Measured specific permeability values and (**b**) diffusion of FITC-dextran 70 kDa particles from the scaffolds to the growth medium over 24 h for 444 networks impregnated with fibrin (444_F) and albumin-rich fibrin (444_AF) hydrogels. Bars represent the mean ± standard error for each tested group (*n* = 5 samples from each group). Statistical analysis was conducted by unpaired *t* test, ** *p* < 0.01.

Scaffold transport properties are important for nutrient uptake, gas exchange, and waste removal-factors that are critical for cell growth and survival [49,50]. The specific permeability values of the 444_F and 444_AF fibre networks were quantified using Darcy’s Law for a constant pressure gradient (Figure 4a). It can be seen that the albumin-enriched fibrin scaffolds have slightly higher specific permeability values 3 × 10^−13^ m^2^ compared to the fibrin scaffolds 1.9 × 10^−13^ m^2^ (*p* < 0.01). In order to study the release kinetics of the hydrogel-impregnated networks, the hydrogels were fabricated with entrapped FITC-conjugated dextran. Figure 4b illustrates the cumulative release of dextran from these scaffolds over 24 h of incubation. Here, 444_AF networks showed higher dextran release compared to 444_F networks. The results suggest that the specific permeability and diffusional transport of nutrient, proteins and growth factors may be somewhat improved in the 444_AF compared to 444_F, which will be beneficial for vessel-like formation (by the HUVECs) and ECM production (by the fHObs).

### 3.4. Vascular Analysis Using AngioTool

**Figure 5:**
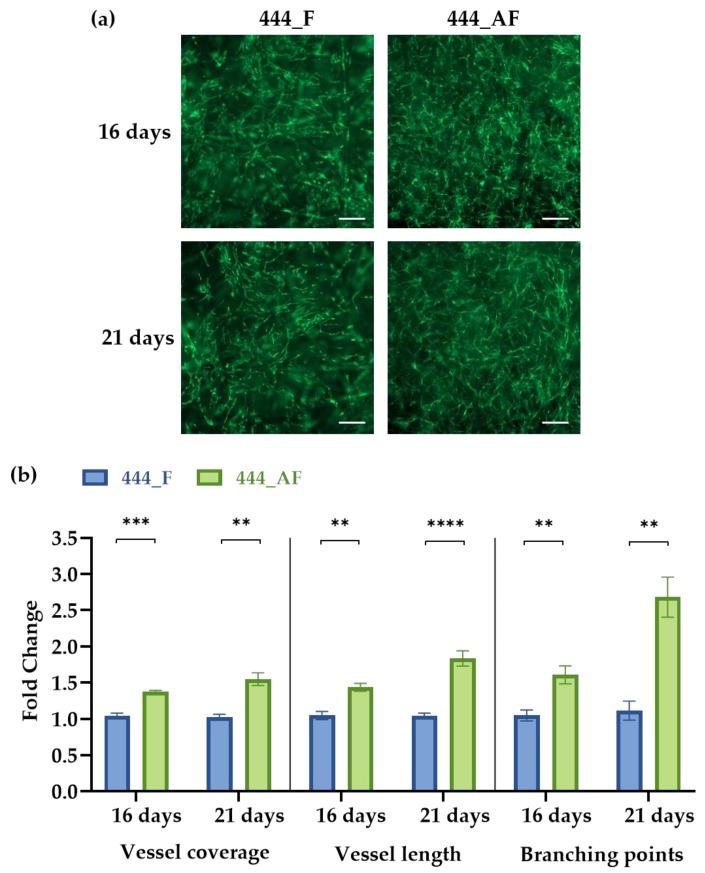
(**a**) Vessel network formation in 444_F and 444_AF scaffolds at days 16 and 21 of culture (scale bar—200 µm); (**b**) Measurements of vessel coverage, total vessel length and total branching points as determined by AngioTool. Data are presented in an x-fold expression of the corresponding 444_F values. Bars represent the mean ± standard error for each tested group (*n* = 3 samples from each group). Statistical analysis was conducted by unpaired *t* test, ** *p* < 0.01, *** *p* < 0.001, **** *p* < 0.0001.

Micro-vessels are essential for bone formation, metabolism, healing and remodelling [15,51]. In vitro vessel-like formations (preliminary endothelial cell structures) in the 444_F and 444_AF scaffolds were imaged using fluorescence microscopy at days 16 and 21 of culture and then analysed using a computational tool for quantitative analysis of vascular network parameters (AngioTool [43]). Vessel coverage and total vessel length were used as a measure of vessel growth, while the number of branch points was used as an indicator of vessel network complexity. Figure 5a shows vessel-like networks in both scaffolds produced by co-cultures of HUVECs and fHObs. It shows that 444_AF had a higher vessel coverage, defined as the area of the given field occupied by vessels, compared to 444_F at both time points (16 days: 1.4-fold, *p* < 0.001; 21 days: 1.6-fold, *p* < 0.01). Examination of total vessel length, defined as the length of the vessel per image field, further supported the finding from the vessel coverage with an increase of vessel length for the 444_AF (16 days: 1.5-fold, *p* < 0.01; 21 days: 1.9-fold, *p* < 0.0001). In addition, 444_AF networks were found to contain more branch points than 444_F (16 days: 1.6-fold, *p* < 0.01; 21 days: 2.7-fold, *p* < 0.01). The results suggest that vessel formation and branching complexity were enhanced with the addition of albumin.

### 3.5. Cell Mineralisation

**Figure 6:**
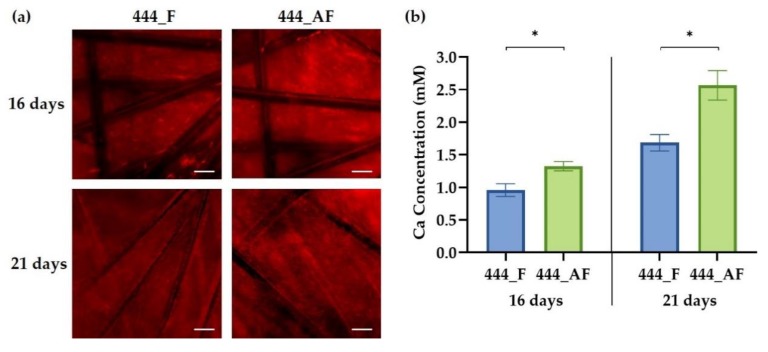
(**a**) Fluorescence imaging of calcium-rich deposits, stained with Alizarin Red, of 444_F and 444_AF scaffolds at days 16 and 21 of culture. Red areas indicate positive staining for calcium-rich deposits (scale bar—100 µm); (**b**) Calcium concentration at days 16 and 21 for the tested groups measured from the released Alizarin red dye using a plate reader at 405 nm. Bars represent the mean ± standard error for each tested group (*n* = 3 samples from each group). Statistical analysis was conducted by unpaired *t* test, * *p* < 0.05.

ECM production was evaluated using Alizarin Red staining for calcium deposition at days 16 and 21 of culture. Figure 6a shows that the osteoblasts undergo osteogenic differentiation by producing mineralised nodules in both scaffolds. The staining revealed a thick and dense calcium-rich (red) layer of new mineral matrix synthesised by the osteoblasts. Figure 6b shows that the calcium concentrations in the 444_AF were significantly higher (*p* < 0.05) compared to the 444_F scaffolds at both time points. This suggests that 444_AF scaffolds can promote mineralisation and ECM deposition. The results are consistent with the findings of Ishida et al. [21], who reported that the presence of albumin in the culture medium caused a significant increase in calcium contents in the femoral-diaphyseal and -metaphyseal tissues obtained from normal rats in vitro. They proposed that albumin stimulates bone formation and albumin plays a role in the regulation of bone metabolism [20,21,22].

It is well known that cell-matrix interaction and ECM deposition play a critical role in vascularisation [42]. Extensive ECM deposition in the 444_AF scaffolds by the osteoblasts provided improved three-dimensional support for HUVECs to migrate and organise into vessel-like structures, as shown in Figure 5.

There are two possible explanations for the above observations. First, the higher specific permeability and protein diffusion (Figure 4) of the 444_AF scaffolds results in an improved mineral deposition by the osteoblasts and vessel formation by the endothelial cells [52,53]. Second, albumin plays an active role in osteoblastic bone formation, though a specific receptor and mechanism have not yet been identified in the literature [22,23].

### 3.6. Quantification of Gene Expression Levels

**Figure 7:**
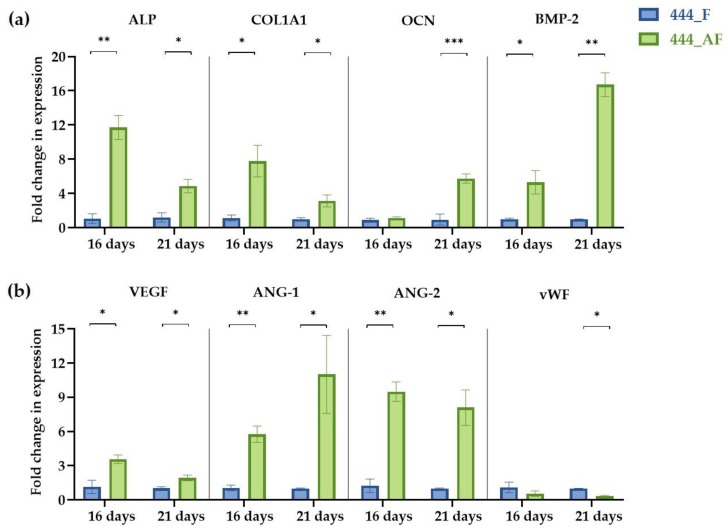
Gene expression of co-cultured HUVECs and fHObs in 444_F and 444_AF obtained by RT-PCR at days 16 and 21 of culture. (**a**) Expression of osteogenic markers: ALP, COL1A1, OCN, BMP-2; (**b**) Expression of angiogenic markers: VEGF, Ang-1, Ang-2, vWF. Data = mean ± standard error (*n* = 3 samples from each group) is reported in *x*-fold expression of the corresponding 444_F values. Statistical analysis was conducted by unpaired *t* test, * *p* < 0.05, ** *p* < 0.01, *** *p* < 0.001. Human alkaline phosphates, ALP; Collagen type 1α1, COL1A1; osteocalcin, OCN; bone morphogenetic protein 2, BMP-2; vascular endothelial growth factor, VEGF; von-Willebrand factor, vWF; angiopoietin 1, Ang-1; angiopoietin 2, Ang-2.

Following the observed increase in vessel formation and calcium deposition for the 444_AF compared to 444_F, the expression of four osteogenic genes (ALP, COL1A1, OCN, and BMP-2) and four angiogenic genes (VEGF, vWF, Ang-1, and Ang-2) at days 16 and 21 of culture were examined using RT-PCR (Figure 7). It can be seen that the expression levels of the osteogenic genes (Figure 7a), ALP and COL1A1 for 444_AF scaffolds were significantly higher than that of 444_F, at both time points, with the highest fold-changes observed at day 16 (ALP: 12-fold and COL1A1: 8-fold). COL1A1 is the main component in the osseous extracellular matrix. It can mediate cell adhesion, provide a template for mineralisation, and drive endothelial cell migration [16,54]. ALP is an ectoenzyme, highly expressed in active osteoblasts [55], which and plays a role in bone mineralisation by controlling the concentrations of mineralisation inhibitors and phosphate ions. Therefore, the high expression values of these genes and the higher values of calcium deposition from the mineralisation assay (Figure 6) suggests that the higher mineral deposition observed in 444_AF compared to 444_F provided an improved 3D support for HUVECs to migrate and organise into vessel-like structures (Figure 5). In the case of OCN and BMP-2, the expression levels for 444_AF were also elevated compared to 444_F at both time points, with the highest fold-changes at day 21 of culture (OCN: 6-fold and BMP-2: 17-fold). OCN is the second most abundant protein in bone after collagen that can be found in a fully mineralised matrix (late markers of osteoblast differentiation) and promotes deposition of mineral substance [56]. BMP-2 accumulates in ECM and has been shown to stimulate osteoblastic differentiation in vitro [57]. It exhibits this osteogenic action by regulating transcription of osteogenic genes such as ALP, COL1A1 and OCN. The above results demonstrate that the addition of albumin to the fibrin hydrogel could promote osteogenesis.

With regards to angiogenic expression, VEGF, Ang-1 and Ang-2 were significantly upregulated in 444_AF compared to 444_F at both time points (Figure 7b). Since VEGF is a potent pro-angiogenic factor with well-established actions on endothelial cells [16], the higher VEGF expression in 444_AF contributed to the better formation of vessel-like structures as shown in Figure 5. Furthermore, the synergy between BMP-2 and VEGF has been reported [58], in which there is an intimate relation to bone development and healing that is advantageous for bone regeneration procedures. In 444_AF, the higher expression of Ang-1, which has an important role in maintaining vessel quiescence [59], and Ang-2, which is a vessel destabiliser [60] with an important role in vascular remodelling, may indicate that these vessels reached higher maturation levels and thus are stable over longer time periods. In both scaffolds, vWF gradually decreased from day 16 to 21 with lower expression levels for 444_AF (16 days: 0.5-fold, 21 days: 0.3-fold); however, significant differences between these groups were only detected at day 21 (*p* < 0.05). vWF is a large multimeric glycoprotein present in blood plasma with multiple roles in vascular development [61], it is required for normal haemostasis, and a decreased expression of vWF increases angiogenesis and vessel formation [16]. Therefore, the decreased expression of vWF with increasing time in the present study is consistent with the observed increase in vascularisation. The fact that vWF at day 21 was lower for 444_AF than 444_F is also consistent with more extensive vessel-like structure formation in 444_AF than 444_F as shown in Figure 5. These results demonstrate that the addition of albumin to the fibrin hydrogel could promote angiogenesis.

In summary, this study shows that albumin can be successfully incorporated in fibrin hydrogel. The addition of 10 μg·mL^−1^ human albumin to the fibrin hydrogel improves the specific permeability and diffusional characteristics (Figure 4) without affecting the Young’s modulus of the hydrogels (Figure 3). Since effective permeability and diffusion of mass transport, as well as sufficient matrix stiffness to support the cells, are essential features of three-dimensional porous scaffolds, the albumin-enriched hydrogel shows a greater potential to serve as a bio-matrix for bone regeneration compared to fibrin alone.

When examined in vitro using co-culture of endothelial cells and osteoblasts, the beneficial effects of albumin-enriched hydrogels on the osteogenesis and angiogenesis were clearly illustrated by upregulation of gene and growth factors expression (Figure 7). Furthermore, the mineralisation production and deposition by the osteoblasts (Figure 6) and the vessel-like structures by the endothelial cells (Figure 5) were augmented with the presence of albumin, reinforcing the important role of albumin plays in bone formation and regulation of bone metabolism.

Future work will focus on the effect of magneto-mechanical actuation on the osteoblast differentiation and vascular self-organisation in 444 ferromagnetic fibre networks impregnated with albumin-enriched hydrogels. Also, an investigation will be carried out using different concentrations of albumin in fibrin hydrogels and on the pathways linked to albumin receptors in osteoblast and endothelial cells.

## 4. Conclusions

This study showed for the first time that albumin-enriched fibrin hydrogel embedded in 444 ferromagnetic fibre network improved extracellular matrix deposition by the osteoblasts and vascularisation by the endothelial cells. Furthermore, this enriched hydrogel promotes osteogenesis by upregulating ALP, COL1A1, OCN, and BMP-2, and angiogenesis by upregulating Ang-1, Ang-2, VEGF, and vWF compared to fibrin hydrogel alone. Albumin was found to increase the specific permeability and diffusional characteristics of the fibrin hydrogels without affecting their stiffness. The results support the potential of this novel albumin-enriched fibrin hydrogel in bone tissue engineering and orthopaedic applications.

## Figures and Tables

**Table 1:** Primer sequences for RT-PCR.

Gene	Oligo Name	Primer Sequence
GAPDH	hum_GAPDH_5/3	5: CTCTGCTCCTCCTGTTCGACA 3: ACGACCAAATCCGTTGACTC
ALP	hum_ALP_5/3	5: CCCAAAGGCTTCTTCTTG 3: CTGGTAGTTGTTGTGAGCAT
OCN	hum_OCN_5/3	5: GACTGTGACGAGTTGGCTGA 3: CTGGAGAGGAGCAGAACTGG
COL1A1	hum_COL1A1_5/3	5: ATGCCTGGTGAACGTGGT 3: AGGAGAGCCATCAGCACCT
vWF	hum_vWF_5/3	5: CGGCTTGCACCATTCAGCTA 3: TGCAGAAGTGAGTATCACAGCCATC
VEGFA	HM_VEGFA_SLFW_Fwd/Rev	F: GAGCCTTGCCTTGCTGCTCTAC R: CACCAGGGTCTCGATTGGATG
ANGPT1	HM_ANGPT1_SLFW_Fwd/Rev	F: CCTGATCTTACACGGTGCTGATT R: GTCCCGCAGTATAGAACATTCCA
ANGPT2	HM_ANGPT2_SLFW_Fwd/Rev	F: AAGAGATCAAGGCCTACTGTGACA R: TCCTCACGTCGCTGAATAATTG
